# Prolactin regulates H3K9ac and H3K9me2 epigenetic marks and miRNAs expression in bovine mammary epithelial cells challenged with *Staphylococcus aureus*

**DOI:** 10.3389/fmicb.2022.990478

**Published:** 2022-09-23

**Authors:** Marco Antonio Barajas-Mendiola, María Guadalupe Salgado-Lora, Joel Edmundo López-Meza, Alejandra Ochoa-Zarzosa

**Affiliations:** Centro Multidisciplinario de Estudios en Biotecnología, Facultad de Medicina Veterinaria y Zootecnia, Universidad Michoacana de San Nicolás de Hidalgo, Morelia, Mexico

**Keywords:** prolactin, innate immunity, epigenetic, mastitis, *Staphylococcus aureus*

## Abstract

Epigenetic mechanisms are essential in the regulation of immune response during infections. Changes in the levels of reproductive hormones, such as prolactin, compromise the mammary gland’s innate immune response (IIR); however, its effect on epigenetic marks is poorly known. This work explored the epigenetic regulation induced by bovine prolactin (bPRL) on bovine mammary epithelial cells (bMECs) challenged with *Staphylococcus aureus*. In this work, bMECs were treated as follows: (1) control cells without any treatment, (2) bMECs treated with bPRL (5 ng/ml) at different times (12 or 24 h), (3) bMECs challenged with *S. aureus* for 2 h, and (4) bMECs treated with bPRL at different times (12 or 24 h), and then challenged with *S. aureus* 2 h. By western blot analyses of histones, we determined that the H3K9ac mark decreased (20%) in bMECs treated with bPRL (12 h) and challenged with *S. aureus*, while the H3K9me2 mark was increased (50%) in the same conditions. Also, this result coincided with an increase (2.3-fold) in HDAC activity analyzed using the cellular histone deacetylase fluorescent kit FLUOR DE LYS^®^. ChIP-qPCRs were performed to determine if the epigenetic marks detected in the histones correlate with enriched marks in the promoter regions of inflammatory genes associated with the *S. aureus* challenge. The H3K9ac mark was enriched in the promoter region of *IL-1β*, *IL-10*, and *BNBD10* genes (1.5, 2.5, 7.5-fold, respectively) in bMECs treated with bPRL, but in bMECs challenged with *S. aureus* it was reduced. Besides, the H3K9me2 mark was enriched in the promoter region of *IL-1β* and *IL-10* genes (3.5 and 2.5-fold, respectively) in bMECs challenged with *S. aureus* but was inhibited by bPRL. Additionally, the expression of several miRNAs was analyzed by qPCR. Let-7a-5p, miR-21a, miR-30b, miR-155, and miR-7863 miRNAs were up-regulated (2, 1.5, 10, 1.5, 3.9-fold, respectively) in bMECs challenged with *S. aureus*; however, bPRL induced a down-regulation in the expression of these miRNAs. In conclusion, bPRL induces epigenetic regulation on specific IIR elements, allowing *S. aureus* to persist and evade the host immune response.

## Introduction

Bovine mastitis is an inflammatory disease of the mammary gland that generates significant economic loss to dairy farming worldwide due to reduced milk quality, high treatment costs due to the infection, decrease in milk production, fertility problems, or the cull of animals with chronic mastitis (Huijps et al., 2008; Reshi et al., 2015; Ruegg, 2017; Dego, 2020). This disease usually results from microbial infections, and *Staphylococcus aureus* is the most prevalent causal pathogen in many regions (9–17% from milk samples) (Wilson et al., 1997; Pitkälä et al., 2004). In addition, *S. aureus* is isolated more frequently from subclinical mastitis than clinical mastitis (up to 75% of the cases in US herds) (Dego, 2020). This pathogen can internalize into the mammary gland’s epithelial cells, evading the immune system, favoring chronic infections, and failure in antibiotic therapy (Barkema et al., 2006; Atalla et al., 2008; Barlow, 2011; Conlon, 2014; Josse et al., 2017; Lee et al., 2018; Zaatout et al., 2020).

Epigenetic mechanisms (for example, chemical modifications on the DNA and histones) have a relevant role in the regulation of differentiation, function, and health of the mammary gland (Devinoy and Rijnkels, 2010; Ivanova et al., 2021; Wang and Ibeagha-Awemu, 2021). For example, in bovines, the hypomethylation of α-casein gene favors its expression, while methylation represses its expression (Singh et al., 2010). Also, there is an increase in the DNA methylation in peripheral blood lymphocytes isolated from cows with mastitis naturally infected with *S. aureus* (Song et al., 2016). Besides, the histone H3K27me3 mark was up-regulated in isolated peripheral blood lymphocytes from cows with mastitis caused by *S. aureus.* It was related to a down-regulation in the expression of the inflammatory response genes (He et al., 2016). Recently, our research group reported that combined bovine prolactin (bPRL) and 17β-estradiol modulate the IIR during *S. aureus* infection using an *in vitro* model of bovine mastitis, and these effects were related to the regulation of H3 histone modifications, such as H3K9ac and H3K9me2 (Salgado-Lora et al., 2020).

The epigenetic regulation also may be achieved through micro RNAs (miRNAs), and several reports evidence its role in bovine bacterial infections (Do et al., 2021). For example, a miRNA transcriptional analysis of MAC-T cell line challenged with *S. aureus* and *Escherichia coli* revealed a single and differential pattern of miRNAs expression, where bta-miR-184, miR-24-3p, miR-148, mir-486, and Let-7a-5p were unique miRNAs expressed during *E. coli* infection, while bta-miR-2339, miR-499, miR-99a, and miR.23a only were expressed during *S. aureus* infection (Jin et al., 2014). In addition, the miRNA expression analysis of monocytes-derived macrophages infected with *Streptococcus agalactiae* showed that miRNAs promote an inflammatory phenotype in these cells regulating the expression of genes coding for *IL-6, IL-1β*, and *TNF-α* (Lewandowska-Sabat et al., 2018).

Mammary gland function is regulated by hormones (for example, prolactin, 17β-estradiol, progesterone, glucocorticoids). Abrupt hormone levels change during the peripartum and lactation has been associated with the risk of mastitis development due to an impaired IIR (Lamote et al., 2006; Brisken and O’Malley, 2010; Lacasse et al., 2019; Alhussien and Dang, 2020). Prolactin is a hormone of 23 kDa (199 amino acids) synthesized mainly by the lactotrophic cells of the anterior pituitary gland with a relevant role in lactation regulation; however, this hormone also is involved in the regulation of the immune response (Binart, 2017; Savino, 2017; Berczi and Nagy, 2019).

Previously, we reported that bPRL at physiological concentrations (5 ng/ml) induces the internalization of *S. aureus* into bovine mammary epithelial cells (bMECs) and modulates the IIR of these cells. Particularly, bPRL up-regulated the expression of *TNF-α, IL-1β*, and *IL-6* genes; however, cells pre-treated with this hormone inhibited the bacterial induction of these genes. Similarly, the expression of the anti-inflammatory cytokine *IL-10* gene was induced by bPRL (Medina-Estrada et al., 2015). Despite the epigenetic role of bPRL on the mammary gland and IIR, their effects on specific epigenetic modifications in the promoter region of IIR genes and miRNAs expression in intramammary infections caused by *S. aureus* are scarce. Thus, this work aimed to characterize if the effects of bPRL on IIR genes in bMECs occur through epigenetic modifications of the promoter region of inflammatory response genes. Besides we evaluate if other elements, such as miRNA expression, were modulated by bPRL in cells challenged with *S. aureus*. This information is relevant to knowing the epigenetic mechanisms underlying bovine mastitis, considering that bPRL receptor signal pathways have been associated with this disease (Khan et al., 2020).

## Materials and methods

### Bovine prolactin

Purified bovine prolactin (bPRL) (AFP7170E) was provided by A. F. Parlow from National Hormone & Peptide Program (NHPP)-National Institute of Diabetes and Digestive and Kidney Diseases (NIDDK) (Torrance, CA, USA) (Parlow, 2002). The hormone was dissolved in sterile water. We used 5 ng/ml in all experiments, as reported (Gutiérrez-Barroso et al., 2008).

### Antibodies

For western blot assays, mouse monoclonal antibody anti-H3K9Ac (1:2,000) (Sc36616, Santa Cruz, CA, USA) and mouse monoclonal antibody anti-H3K9me2 ChIP grade (1:1,000) (ab1220, Abcam, Cambridge, UK) were used as primary antibodies. The rabbit polyclonal antibody anti-H3-ChIP grade (1:5,000) (ab1791, Abcam, Cambridge, UK) was used as load control. Secondary antibodies (1:3,000) raised against mouse and rabbit antibodies coupled to radish peroxidase were purchased from Cell Signaling Technology (Danvers, MA, USA).

For the chromatin immunoprecipitation assay (ChIP), 3 μg of the mouse monoclonal antibody anti-H3K9me2-ChIP grade (ab1220, Abcam, Cambridge, UK) and rabbit polyclonal antibody anti-H3K9ac-grade ChIP (ab10812, Abcam, Cambridge, UK) were used in each assay. In addition, rabbit polyclonal antibody anti-H3-grade ChIP (Abcam, ab1791) and the mouse polyclonal antibody anti-IgG (Sigma-Aldrich, St. Louis, MO, USA) were used as reference control and negative isotype control, respectively.

### *Staphylococcus aureus* strain

*Staphylococcus aureus* subsp. *aureus* Rosenbach (ATCC 27543) strain was used in this work. This strain was isolated from a case of clinic mastitis and can invade bovine mammary epithelial cells (Medina-Estrada et al., 2015). Bacteria were grown overnight in Luria-Bertani (LB) broth (BIOXON, Becton Dickinson-México, México) at 37°C. Colony-forming units (CFU) were adjusted by measuring the optical density at 600 nm (OD 0.2 = 9.2 × 10^7^ CFU/ml).

### Primary bovine mammary epithelial cell culture

Bovine mammary epithelial cell (bMECs) were isolated from alveolar tissue of the udder of healthy lactating cows (slaughtered for meat), according to Anaya-López et al. (2006). Cells from passages 2–8 were used in all the experiments. Cells were grown on 90 × 15 mm culture Petri dishes (NEST Biotechnology Co. Wuxi, Jiangsu, China) in Dulbecco’s Modified Eagle’s Medium/Nutrient Mixture F-12 Ham (DMEM/F12-Ham) (Sigma-Aldrich, St. Louis, MO, USA) supplemented with 10% of fetal bovine serum (FBS) (Equitech Bio, Kerrville, TX, USA), insulin 10 μg/ml (Sigma-Aldrich, St. Louis, MO, USA), hydrocortisone 5 μg/ml (Sigma-Aldrich, St. Louis, MO, USA), penicillin 100 U/ml (Gibco, Waltham, MA, USA) streptomycin 100 μg/ml (Gibco) and amphotericin B 1 μg/ml (Sigma Sigma-Aldrich, St. Louis, MO, USA) to obtain the complete medium. Prior to the challenge, cells were maintained in a 5% CO_2_ atmosphere at 37°C. All the experiments were performed using cells synchronized in DMEM/F12-Ham medium without FBS and antibiotics (incomplete medium) for 24 h. bMECs were treated as follows: (1) control cells without any treatment, (2) bMECs treated with bPRL (5 ng/ml) at different times (12 or 24 h), (3) bMECs challenged with *S. aureus* for 2 h, and (4) bMECs treated with bPRL at different times (12 or 24 h), and then challenged with *S. aureus* 2 h. The interaction of *S. aureus* with bMECs for 2 h is based on previous studies from our research group (Anaya-López et al., 2006; Gutiérrez-Barroso et al., 2008; Medina-Estrada et al., 2015), which analyzed the IIR to *S. aureus* challenge after this time.

### Invasion assays

For ChIP assays, monolayers of 10 × 10^6^ bMECs were grown in complete medium on 60 × 15 mm cell culture Petri dishes (NEST Biotechnology Co. Wuxi, Jiangsu, China) previously coated with 500 μl of type 1 rat collagen (Sigma-Aldrich St. Louis, MO, USA). Cells were incubated until reaching 90% of confluence in a 5% CO_2_ atmosphere at 37°C for 4 days. For the miRNAs expression assays, monolayers of 2.5 × 10^5^ bMECs were grown in complete medium on 24-well plates (NEST Biotechnology Co., Wuxi, Jiangsu, China) previously coated with 200 μl of type 1 rat collagen. Cells were incubated until reaching 90% of confluence in a 5% CO_2_ atmosphere at 37°C for 24 h. Previous to the challenge, cells were synchronized with the incomplete medium in a 5% CO_2_ atmosphere at 37°C for 24 h. Then, cells were treated with bPRL (5 ng/ml) and incubated in a 5% CO_2_ atmosphere at 37°C for 12 h or 24 h. The invasion assay was performed using the gentamicin protection assay previously described (Gutiérrez-Barroso et al., 2008; Alva-Murillo et al., 2014). Briefly, bMECs treated with bPRL were challenged with *S. aureus* (OD = 0.2) at a multiplicity of infection (MOI) of 30:1 (bacteria: cell) in a 5% CO_2_ atmosphere at 37°C for 2 h. Later, cells were washed three times with PBS (pH 7.4), and an incomplete medium supplemented with gentamicin 80 μg/ml was added to eliminate extracellular bacteria for 1 h. Finally, cells were washed three times with PBS (pH 7.4) and were employed for ChIP and miRNAs expression assays as indicated below.

### Chromatin immunoprecipitation-qPCR assays

Enrichment of the epigenetic marks on the promoter region of the IIR genes was assessed by ChIP, as described by Carey et al. (2009), with some modifications. DNA-protein complexes of the bMECs (10 × 10^6^ cells) were fixed by cross-linking with 1% of formaldehyde (Baker Analyzed, J. T. Baker) in agitation for 10 min, and the reaction was stopped with glycine 1.35 M (Sigma) for 5 min. Then, cells were washed three times with cold PBS (pH 7.4), detached with a sterile cell scraper (Corning, Manassas, VA, USA), and centrifuged at 1,500 rpm for 10 min at 4°C. The cell pellet was resuspended in 10 ml of cold lysis buffer for ChIP (50 mM HEPES-KOH pH 7.5, 140 mM NaCl, 1% Triton X-100, 0.1% sodium deoxycholate, 0.1% SDS) and incubated 10 min on ice. The nuclei were pelleted at 1,500 rpm for 10 min at 4°C, then resuspended in cold lysis buffer with 10 μl of a cocktail of protease inhibitors (Sigma-Aldrich St. Louis, MO, USA). The chromatin was sheared by sonication (6 × 15 s pulses followed by 60 s rest). 300 μl chromatin (100 μg/ml) diluted in cold dilution buffer for ChIP (1% Triton X-100, 2 mM EDTA pH 8, 20 mM Tris-HCl pH 8, 150 mM NaCl) were supplemented with 3 μl of a cocktail of protease inhibitors and 50 μl of a mixture of protein A agarose/protein G agarose beads (Invitrogen, Waltham, MA, USA) were added to preclear the chromatin in agitation at 4°C for 2 h. Next, the samples were centrifuged at 3,000 rpm for 5 min at 4°C, the supernatants were transferred to new microtubes, and 3 μg of the specific antibody was added and maintained in agitation overnight at 4°C. Again, 50 μl of a mixture of protein A agarose/protein G agarose beads were added to each sample and kept in agitation at 4°C for 2 h. Then, the samples were centrifuged at 3,000 rpm for 3 min at 4°C, and the supernatants were removed carefully. Further, 1 ml of cold wash buffer for ChIP (0.1% SDS, 1% Triton X-100, 2 mM EDTA pH 8, 20 mM Tris-HCl pH 8, 150 mM NaCl) was added to samples, maintained in agitation for 10 min at room temperature, and centrifuged at 3,000 rpm for 3 min at 4°C, three times. Then, 1 ml of cold final wash buffer for ChIP (0.1% SDS, 1% Triton X-100, 2 mM EDTA pH 8, 20 mM Tris-HCl pH 8, 500 mM NaCl) was added, maintained in agitation for 10 min at room temperature, and centrifuged at 3,000 rpm for 3 min at 4°C. The beads were resuspended in 300 μl of elution buffer for ChIP (1% SDS, 100 mM NaHCO_3_) supplemented with 0.5 μl of proteinase K (20 μg/μl, Thermo Scientific, Waltham, MA, USA) and incubated at 55°C for 2 h. An additional incubation at 65°C for 4 h was performed to reverse the cross-linking. Finally, the samples were incubated for 5 min at room temperature and centrifuged at 13,000 rpm for 5 min. The supernatants were transferred to new microtubes, and the DNA was isolated by phenol/chloroform extraction and frozen at –80°C until use. The enrichment of epigenetic marks was analyzed by quantitative PCR using the comparative Ct method (ΔΔCt) in a StepOne Plus Real-Time PCR System (Applied Biosystems, Waltham, MA, USA) according to the manufacturer’s instructions. The reactions were carried out with qPCRBIO SyGreen Blue Mix Hi-ROX master mix (Biosystems, Wayne, PA, USA) with specific primers (Invitrogen, Waltham, MA, USA) for the promoter region of the IIR genes. The primer sequences and the PCR conditions used to amplify the promoter region of the bovine IIR genes are described in Table 1. GAPDH was used as a positive control for the analysis of H3K9ac enrichment and negative control for studying enrichment in H3K9me2.

**TABLE 1:** Primers used for the analysis of the promoter region of innate immune response genes.

Bovine (genes)^1^	Sequence (5′→3′)	Tm (°C)	Fragment (bp)	Reference
TNF-α	F-GCTCATGGGTTTCTCCACCCA	60	197	This work
	R-GGAGGTTATCTCCAGGGGGT			
IL-1β	F-TTGCCTGCCAGGTACAAGAT	60	154	This work
	R-ACCATTTGCCATGCCAGTCC			
IL-8	F-CAAAGCTTGGGTCACACAG	60	183	This work
	R-AGGGAACAAGTGCACCATC			
IL-10	F-GCGAAGGTTCAGCAAGAAG	60	129	This work
	R-TAATGAGGCTGGGGAAAACC			
LAP	F-GCTGAACTGACTGCCAGGAA	58.5	125	This work
	R-GGAGTGGCCTTCATAGCACA			
BNBD10	F-CAGGGGAAACAGGCAAGTCT	55.5	147	This work
	R-GGGGCAGTCACTTGTATGCT			
GAPDH	F-CTCTAATGTTCACCTTCCTC	58	191	This work
	R-CGACCACCCTATTCAGGTTC			

^1^TNF-α, tumor necrosis factor-alpha; IL-1β, interleukin 1-beta; IL-8, chemokine interleukin 8; IL-10, interleukin 10; LAP, lingual antimicrobial peptide; BNBD10, bovine neutrophil beta-defensin 10; GAPDH, glyceraldehyde 3-phosphate dehydrogenase.

### RNA isolation and miRNAs expression

To analyze the effect of bPRL and *S. aureus* on the miRNAs expression, monolayers of 2.5 × 10^5^ cells were cultured in 24-well plates (NEST, Biotechnology Co., Wuxi, Jiangsu, China) and incubated with bPRL (5 ng/ml) for 12 h and challenged or not with *S. aureus* (MOI 30:1). The total RNA was extracted using the Trizol™Reagent (Invitrogen, Waltham, MA, USA) according to the manufacturer’s instructions. The integrity of total RNA was verified by agarose gel electrophoresis, and the genomic DNA contamination was removed with DNAse I (Invitrogen, Waltham, MA, USA) according to the manufacturer’s instructions. Then, the cDNA was synthesized according to Varkonyi-Gasic and Hellens (2011), with some modifications. Briefly, 1 μg of total RNA was reversed transcribed to cDNA using the M-MLV-Reverse Transcriptase (Invitrogen, Waltham, MA, USA) in a reaction of 20 μl containing 1 μM stem-loop RT primer and 10 μM dNTPs mix (Invitrogen, Waltham, MA, USA). The reaction was incubated at 65°C for 5 min and then transferred to ice. Further, 1X First-Strand Buffer, 10 mM dithiothreitol, and 2 U/μl of RNAseOUT™ (Invitrogen, Waltham, MA, USA) were added to the reaction mixture incubated at 37°C for 2 min. Finally, 5 U/μl of M-MLV reverse transcriptase was added, and the reaction mixture was incubated at 37°C for 50 min, followed by 70°C for 15 min. The RT-qPCR assay was performed by quantitative PCR using the comparative Ct method (ΔΔCt) in a StepOne Plus Real-Time PCR System (Applied Biosystems, Waltham, MA, USA) according to the manufacturer’s instructions. The reactions were carried out with qPCRBIO SyGreen Blue Mix Hi-ROX master mix (Biosystems, Wayne, PA, USA) with the specific primers (Elim Biopharm, Hayward, CA, USA) for the miRNAs. The stem-loop primer and the sequences of the primers used to amplify bovine miRNAs were designed using the miRNA Primer Design Tool^1^. The sequences and the PCR conditions used to amplify the bovine miRNAs are described in Table 2. U6 miRNA was used as an internal control of amplification.

**TABLE 2:** Primers used for the analysis of bovine miRNA expression.

Bovine miRNA		Sequence 5′ to 3′	Tm (°C)	Reference
Let-7a-5p	^a^Stem-Loop	GTTGGCTCTGGTGCAGGGTCCGAGG TATTCGCACCAGAGCCAAC	54	This study
	^b^Universal Reverse Primer	GTGCAGGGTCCGAGGT		
	^c^miRNA specific sequence	AACTAT		
	^d^Forward Primer	GGGTGAGGTAGTAGGTTGT		
miR-21a	^c^miRNA specific sequence	AGTCAA	56	This study
	^d^Forward Primer	GTTGTAGCTTATCAGACTGATG		
miR-30b	^c^miRNA specific sequence	AGCTGA	60	This study
	^d^Forward Primer	GTTTGGTGTAAACATCCTACAC		
miR-155	^c^miRNA specific sequence	ACCCCT	56	This study
	^d^Forward Primer	GTGGGTTAATGCTAATCGTGAT		
miR-23a	^c^miRNA specific sequence	TGGAAA	58	This study
	^d^Forward Primer	GTGATCACATTGCCAGGGA		
miR-7863	^c^miRNA specific sequence	TGGAAA	50	This study
	^d^Forward Primer	GTTGATGGACTGTCACCTG		
miR-146a	^c^miRNA specific sequence	ACAACC	52	This study
	*^d^*Forward Primer	GTGGTGAGAACTGAATTCCATA		
miR-144	^c^miRNA specific sequence	ACAACC	58	This study
	*^d^*Forward Primer	GTTGGGTACAGTATAGATGATG		
miR-451	^c^miRNA specific sequence	AAACTC	52	This study
	^d^Forward Primer	GTTGGAAACCGTTACCATTACT		
U6	Stem-Loop	CGCTTCACGAATTTGCGTGTCAT	50 to 60	(Han et al., 2020)
	Reverse Primer	GCTTCGGCAGCACATATACTAAAAT		
	Forward Primer	CGCTTCACGAATTTGCGTGTCAT		

^a^The Stem-Loop and the ^b^Universal Reverse Primer sequences are the same for all miRNAs evaluated except for the ^c^miRNA specific and ^d^forward primer sequences. Primers were designed using the miRNA Primer Design Tool date base [miRNA Primer Design Tool | Login (dote.hu)].

### Extraction and purification of histones

To analyze the effects of bPRL on the global epigenetics marks H3K9ac and H3K9me2 of bMECs, the histones were extracted according to the acidic extraction for histones protocol described by Shechter et al. (2007) and modified by Salgado-Lora et al. (2020). Briefly, 3 × 10^6^ cells were cultured in Petri dishes at 90% of confluence, then bPRL (5 ng/ml) was added for 12 or 24 h, and then the cells were challenged with *S. aureus* for 2 h. Next, the cells were washed three times with PBS (pH 7.4), detached with trypsin (0.05%)-EDTA (0.02%) (Sigma-Aldrich, St. Louis, MO, USA), and centrifuged at 3,200 rpm at 4°C for 10 min. Next, the pellet was washed with PBS (pH 7.4) and resuspended in 1 ml of hypotonic lysis buffer (10 mM Tris-HCl pH 8.0, 1 mM KCl, 1.5 mM MgCl_2_, 1 mM DTT), and cells were maintained in agitation at 4°C for 30 min. After, the intact nuclei were pelleted at 10,000 rpm for 10 min at 4°C, resuspended in 400 μl of 0.4 N H_2_SO_4_, and maintained in agitation overnight at 4°C. Next, the samples were centrifuged at 13,200 rpm for 10 min at 4°C; then, histones were precipitated with TCA (33%) and maintained in agitation at 4°C overnight. Next, histones were pelleted at 13,200 rpm for 10 min at 4°C, washed with cold acetone, and centrifugated at 13,200 for 10 min at 4°C; this last step was performed three times. Finally, histones were air-dried for 20 min at room temperature, and the pellet was dissolved in sterile distilled water and stored at –80°C until use. Finally, the histones were resolved on a 15% SDS-PAGE gel to verify the integrity before performing the western blot analysis.

### Western blot assays

Histone proteins were transferred to PVDF membranes using a semi-dry transfer system (Fisher Scientific, Waltham, MA, United States). Membranes were blocked with 5% non-fat milk in PBS (pH 7.4) at 4°C for 4 h and then incubated with primary antibodies: anti-H3ac (1:1,000), anti-H3K9ac (1:200), anti-H3K9me2 (1:1,000), anti-H3 (1:5,000), at 4°C overnight. After this, the primary antibodies were removed, and the membranes were incubated with the mouse or anti-rabbit horseradish peroxidase coupled secondary antibodies (1:3,000) at 4°C for 2 h. Finally, the secondary antibody was removed and ECL western blotting substrate WesternsureT (Thermo Scientific, Waltham, MA, USA) was added and developed using the iBright CL1500 Imaging System (Thermo Scientific, Waltham, MA, USA). Signal intensity was quantified by densitometry using the iBright Analysis software (Thermo Scientific, Waltham, MA, USA). The data of the target histone mark were normalized to H3 expression.

### Histone deacetylases activity assay

Histone deacetylases (HDACs) activity was evaluated using the cellular histone deacetylase fluorescent kit FLUOR DE LYS^®^ (Enzo, Enzo Biochem, New York, NY, USA) according to the manufacturer’s instructions. The kit measures the activity of the HDACs class I (HDAC 1, 2, 3, and 8), class IIa (HDAC 4, 5, 7, and 9), and class IIb (HDAC6 and 10). Briefly, 1 × 10^4^ bMECs were grown on 96-well plates in a complete medium in a 5% CO_2_ atmosphere at 37°C for 24 h. Then, the cells were synchronized for 16 h with the incomplete medium. Cells were treated with bPRL (5 ng/ml) and then were challenged with *S. aureus* (MOI 30:1) for 2 h, as control bMECs without any treatment were used. Next, the supernatant was removed, and 50 μl of acetylated FLUOR DE LYS^®^ substrate was added and incubated at 37°C for 4 h. Later, the substrate was removed, the cells were washed with PBS (pH 7.4) three times, and 25 μl of the developer was added and incubated at 37°C for 5 min. A standard curve was generated using serial dilutions (1:10) of the deacetylated FLUOR DE LYS ^®^ standard and the developer supplied with the kit. Finally, the relative fluorescence units (RFU) of both cells and the standard curve counts were acquired with a Varioskan (Thermo Fisher, Waltham, MA, USA) plate reader (360 nm emission and 460 nm excitation). Trichostatin A (TSA, 1 μM; Sigma-Aldrich, St. Louis, MO, USA) was used as an inhibitor of HDACs activity. The data of HDACs activity was calculated and normalized (to 1-fold) regarding the control condition.

### Histone demethylases activity

Histone demethylases (HDMs) activity was evaluated using the Jumonji family demethylases activity kit (Invitrogen, Waltham, MA, USA). bMECs were grown on 96-well plates and treated as described for the HDACs assay, and cell lysates were obtained according to the manufacturer’s instructions. The product of the enzymatic demethylation reactions is formaldehyde, which was quantitated directly by a fluorescent product with a Varioskan (Thermo Scientific, Waltham, MA, USA) plate reader (450 nm excitation, 510 nm emission). HDM activity was calculated and normalized (to 1-fold) regarding the control condition. According to the manufacturer’s instructions, the assays were run in duplicate from two different experiments.

### Statistics analysis

For western blot and ChIP-qPCR analysis, the data were obtained from at least three and four independent experiments, respectively, in triplicate and were compared with a *t*-student, and one-way analysis of variance (ANOVA) using the *post hoc* Tukey test, respectively. The results are reported as the mean ± standard error (SE) with a significance level of *p* ≤ 0.05, *p* ≤ 0.01, or *p* ≤ 0.001. For HDACs and HDMs activity, the data were obtained from at least three and two independent experiments for duplicated, respectively, and were compared using *t*-student. The results are reported as the mean ± standard error (SE) with a significance level of *p* ≤ 0.05, *p* ≤ 0.01, or *p* ≤ 0.001. Finally, for miRNAs analysis, the data were obtained from at least four experiments in triplicate and were compared with the *t*-student and Wilcoxon test. The results are reported as the mean ± SE with a significance level of *p* ≤ 0.05, *p* ≤ 0.01, or *p* ≤ 0.001.

## Results

### Histone epigenetic marks are regulated by bovine prolactin in bovine mammary epithelial cells during *Staphylococcus aureus* challenge

**FIGURE 1:**
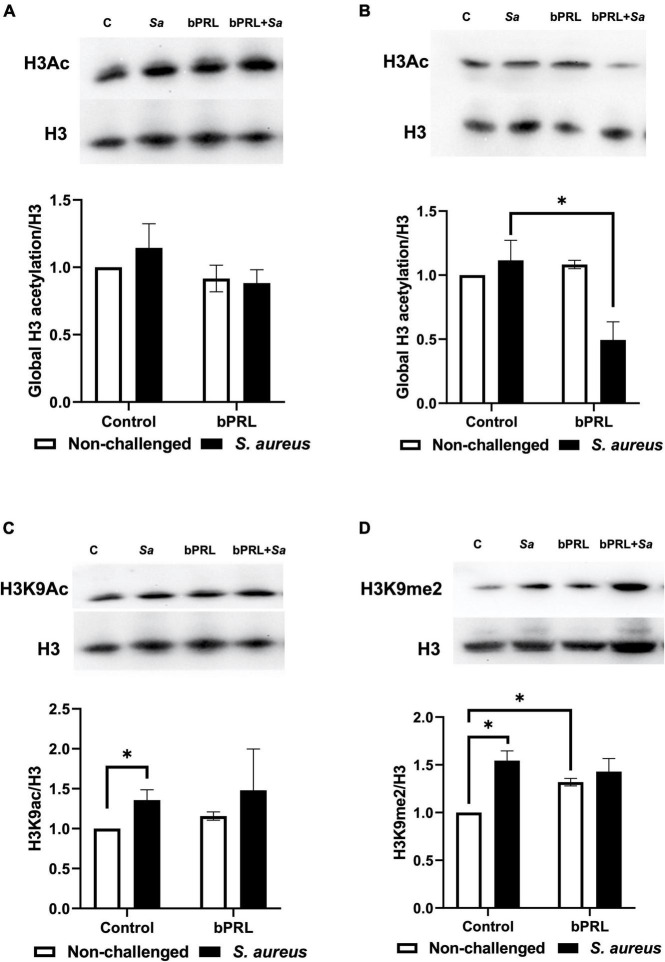
Regulation of histone H3 marks by bPRL in bMECs during *S. aureus* challenge. bMECs were treated with bPRL (5 ng/ml), and cells were or not challenged for 2 h with *S. aureus* (MOI 30:1). Densitometrical analysis of the immunoblots that shows the relative expression of global H3ac regarding the total H3 in bMECs treated 12 h **(A)** and 24 h **(B)** with bPRL and challenged or not challenged. Densitometrical analysis of immunoblots that shows the relative expression of H3K9ac mark **(C)** and H3K9me2 mark **(D)** regarding the total H3 in bMECs treated 12 h with bPRL and challenged or not challenged. C, bMECs without any treatment; bPRL, bMECs treated 12 h or 24 h with bPRL (5 ng/ml); bPRL + *Sa*, bMECs treated 12 or 24 h with bPRL (5 ng/ml) and challenged with *S. aureus* for 2 h (MOI = 30:1); *Sa*, bMECs only challenged with *S. aureus* (MOI = 30:1). Representative western blots are shown above each graph. Bars show the mean ± SE of optical density (arbitrary units, AU), considering the expression of control cells (bMECs without treatment) as 1 (data normalized) obtained from four independent experiments (*n* = 4). The symbol “*” indicates a significant change (*p* ≤ 0.05).

Previously we reported the bPRL effects on IIR of bMECs. In this work, we analyzed the levels of several histone marks to determine if these effects could be related to epigenetic modulation. The results showed that the global acetylation of histone H3 of bMECs was not modified by bPRL at 12 h (Figure 1A). However, in bMECs treated with the hormone for 24 h and challenged with *S. aureus*, the global H3 acetylation decreased by 50% regarding control bMECs (Figure 1B). To determine if the hormone regulates specific H3 residues in bMECs, the H3K9ac and H3K9me2 marks were analyzed. The results showed that cells treated 12 h with bPRL and challenged with *S. aureus* significantly decreased the H3K9ac mark (20%) regarding control (Figure 1C). Interestingly, there were no significant changes in H3K9ac at 24 h (data not shown). Concerning the H3K9me2 mark, bMECs challenged with *S. aureus* increased significantly this mark (50%), as well as cells treated with bPRL (12 h) alone or together with the challenge (30%) (Figure 1D). Finally, bPRL at 24 h of treatment did not modify this mark (data not shown).

**FIGURE 2:**
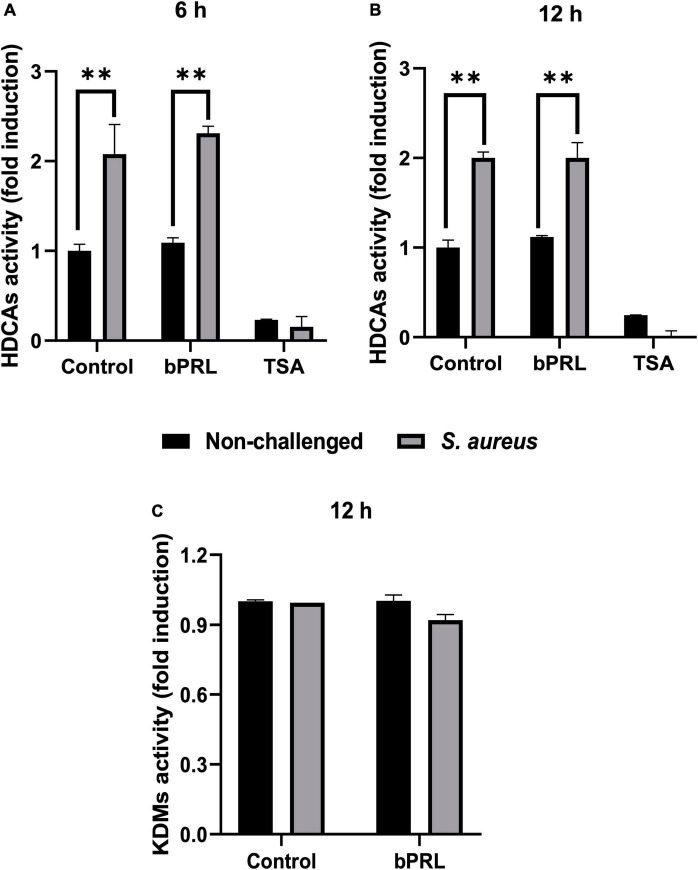
Activity of HDACs and HDMs in bMECs treated with bPRL and challenged with *S. aureus*. bMECs were treated **(A)** 6 and **(B)** 12 h with bPRL (5 ng/ml) and then were challenged or not 2 h with *S. aureus* (MOI 30:1). HDACs substrate was added and incubated according to the manufacturer’s instructions. Total HDACs activity was determined using the HDAC fluorometric cellular activity assay FLUOR DE LYS^®^. Each bar shows the mean ± SE of HDAC activity of cells from three independent experiments for duplicates, considering the expression of control cells (bMECs without treatment) as 1 (data normalized). The symbol “**” (*p* ≤ 0.05) indicates a significant change. Trichostatin (TSA, 1 μM) was used as an inhibitor of HDACs. **(C)** bMECs were treated 12 h with bPRL (5 ng/ml) and then were challenged or not 2 h with *S. aureus* (MOI 30:1), and were incubated with the substrates for Jumonji demethylases (HDMs). Each bar shows the mean of the HDM activity of cell lysates from two different experiments ± SE, which were run in duplicate (*n* = 4), considering the expression of control cells as 1 (data normalized).

To explore if bPRL could be involved in the modulation of H3K9ac through regulating HDACs, the activity of two classes of HDACs (class I and II) was evaluated. The evaluations were performed at 6 and 12 h, considering that the effects of bPRL on the H3K9ac mark were detected at 12 h. Results showed that do not exist significant changes in the HDACs activity in both bMECs treated or not treated with bPRL, but when the bMECs were challenged with *S. aureus*, the HDACs activity was increased (2.3-fold) (Figures 2A,B). Also, we evaluated the HDMs activity (enzymes involved in the methyl group remotion), and the results showed a tendency to reduction in activity (10%) in bMECs treated with bPRL 12 h and challenged with *S. aureus* (Figure 2C); however, there were no significative differences.

### Bovine prolactin regulates the expression of innate immune response elements through H3K9ac and H3K9me2 marks in bovine mammary epithelial cells challenged with *Staphylococcus aureus*

**FIGURE 3:**
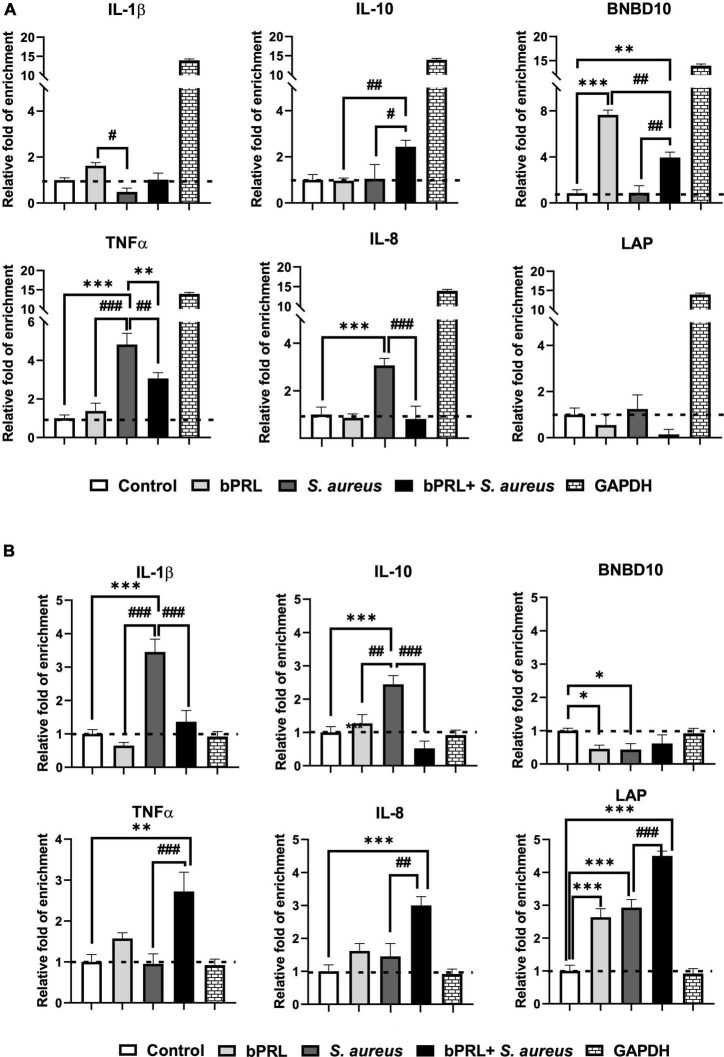
Analysis of epigenetic modulation of H3K9ac and H3K9me2 marks in the promoter region of IIR elements in bMECs treated with bPRL and challenged with *S. aureus*. bMECs were treated 12 h with bPRL (5 ng/ml), and cells were or not challenged for 2 h with *S. aureus* (MOI = 30:1). **(A)** The ChIP-qPCR assay was performed to evaluate the relative enrichment of the H3K9ac mark on the promoter region of *IL-1β*, *IL-10*, *BNBD10*, *TNF-α*, *IL-8*, and *LAP*. **(B)** Relative enrichment of the H3K9me2 mark on the promoter region of *IL-1β*, *IL-10*, *BNBD10*, *TNF-α*, *IL-8*, and *LAP*. Control, bMECs without any treatment; bPRL, bMECs treated 12 h with bPRL (5 ng/ml); bPRL + *S. aureus*: bMECs treated 12 h with bPRL (5 ng/ml) and challenged with *S. aureus* (MOI = 30:1); *S. aureus*, bMECs only challenged with *S. aureus* (MOI = 30:1). The data were normalized regarding IgG-Ab isotype control, and the expression of control cells (cells without treatment) was considered as one. The data correspond to the mean ± SE from four independent experiments (*n* = 4). “*” (*p* ≤ 0.05), “**” (*p* ≤ 0.01), and “***” (*p* ≤ 0.001) indicate a significant change regarding control (bMECs without any treatment). “#” (*p* ≤ 0.05), “##” (*p* ≤ 0.01), and “###” (*p* ≤ 0.001) indicate a significant change regarding bMECs+*S. aureus* condition. GAPDH was used as a positive control of enrichment (for H3K9ac) and not enrichment (for H3K9me2).

As indicated above, the epigenetic marks H3K9ac and H3K9me2 were modified in bMECs treated with bPRL and challenged with *S. aureus*. To determine the role of these marks in the expression of IIR elements regulated by bPRL, ChIP and quantitative qPCR assays were carried out. The results showed that the H3K9ac mark was enriched significantly in the promoter region of *IL-1β, IL-10*, and *BNBD10* genes (1.5, 2.5, and 7.5-fold, respectively) in bMECs treated 12 h with bPRL (Figure 3A). Still, this epigenetic mark was reduced significantly in both bMECs treated with bPRL and challenged and in cells only challenged (Figure 3A). Also, the promoter region of *TNF*-α gene showed an increase of the H3K9ac mark (5-fold) in bMECs challenged; however, in cells challenged and treated with bPRL this epigenetic mark decreased significantly (3-fold) (Figure 3A). Moreover, this mark was not modified in the promoter region of *IL-8* and *LAP* genes under the same conditions (Figure 3A). Additionally, we observed an inverse relationship between the H3K9me2 and H3K9ac marks for the *IL-1β* and *IL-10* genes. H3K9me2 mark was significantly enriched in the promoter region of *IL-1β* and *IL-10* (3.5, 2.5-fold, respectively) in bMECs only challenged. Still, it was reduced in bMECs treated with bPRL and challenged or not challenged (Figure 3B). A similar effect was observed for the *LAP* gene under the same conditions (Figure 3B). Interestingly, the H3K9me2 mark was enriched significantly in the promoter region of *TNF*-α and *IL-8* genes (1.3 and 3-fold, respectively) of bMECs treated with bPRL and challenged (Figure 3B). Altogether, these results indicate that bPRL, through epigenetic modifications mediated by H3K9ac and H3K9me2, allows the regulation of gene expression of IIR elements in bMECs. Moreover, this regulation is modified during *S. aureus* challenge. These marks correlate with the level of the expression of these genes, as reported (Medina-Estrada et al., 2015).

### miRNAs expression in bovine mammary epithelial cells is regulated by bovine prolactin during *Staphylococcus aureus* challenge

**FIGURE 4:**
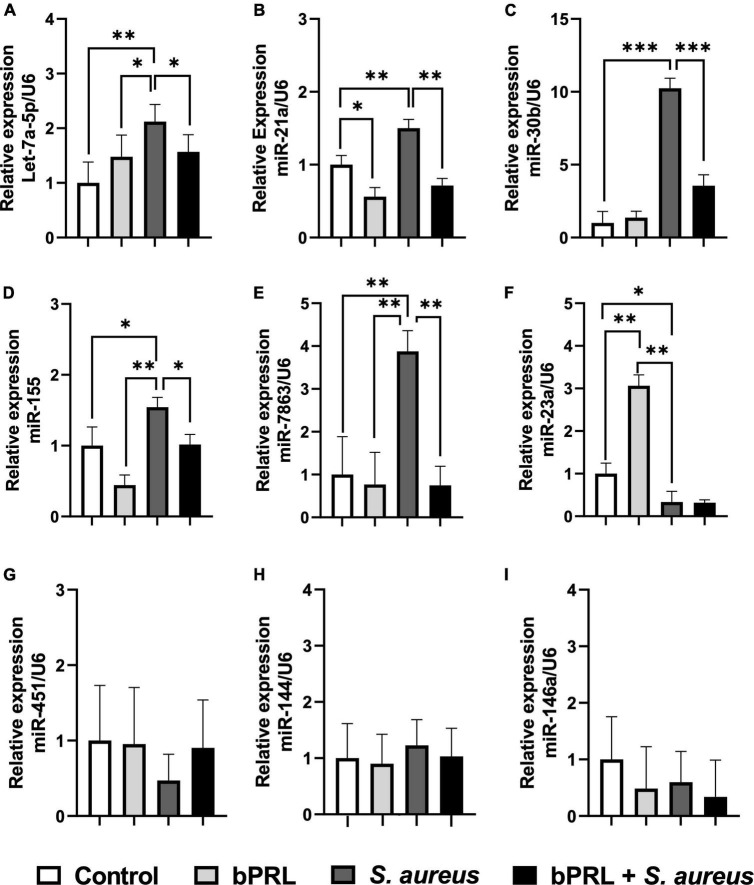
Bovine prolactin regulates the miRNAs expression in bMECs during *S. aureus* challenge. bMECs were treated 12 h with bPRL (5 ng/ml) and then were or not challenged for 2 h with *S. aureus* (MOI = 30:1). The RT-qPCR assay using the ΔΔCt comparative method was performed to evaluate the expression of miRNAs Let-7a-5p **(A)**, miR-21a **(B)**, miR-30b **(C)**, miR-155 **(D)**, miR-7863 **(E)**, miR-23a **(F)**, miR-451 **(G)**, miR-144 **(H)**, miR-146a **(I)**. Control, bMECs without any treatment; bPRL, bMECs treated 12 h with bPRL (5 ng/ml); bPRL + *S. aureus*, bMECs treated 12 h with bPRL (5 ng/ml) and challenged with *S. aureus* for 2 h (MOI = 30:1); *S. aureus*, bMECs only challenged with *S. aureus* (MOI = 30:1). Data were normalized regarding internal control U6. Each bar shows the mean ± SE and was analyzed using *t*-student and Wilcoxon tests from four independent experiments (*n* = 4). “*” (*p* ≤ 0.05), “**” (*p* ≤ 0.01), and “***” (*p* ≤ 0.001) indicate significant differences.

The expression of diverse miRNAs involved in the regulation of immune response of bMECs was evaluated by qPCR in cells treated with bPRL and challenged with *S. aureus*. The results showed an up-regulation in the expression of miRNAs Let-7a-5p, miR-21a, miR-30b, miR-155, and miR-7863 (2, 1.5, 10, 1.5, and 3.9-fold, respectively) in bMECs challenged with *S. aureus* (Figure 4). Also, bPRL (12 h) induced a down-regulation of these miRNAs in bMECs challenged or not challenged (Figure 4). Further, a significant increase in the miR-23a expression (3-fold) was observed in bMECs treated only with bPRL (12 h); however, *S. aureus* induced a significant decrease of this miRNA in bMECs treated or not treated with the hormone (Figure 4F). On the other hand, the expression of miR-451, miR-144, and miR146a miRNAs was not modified (Figures 4G–I). These findings show that bPRL, through the miRNA’s expression, could participate in regulating immune response mounted in bMECs challenged with *S. aureus*.

## Discussion

Bovine mastitis susceptibility has been associated with abrupt changes in the levels of reproductive hormones that induce an impaired immune response (Lamote et al., 2006; Lacasse et al., 2019; Alhussien and Dang, 2020). Previously we showed that bPRL induces a pro-inflammatory response in bMECs, which is down-regulated in cells challenged with *S. aureus* (Ochoa-Zarzosa et al., 2007; Gutiérrez-Barroso et al., 2008; Lara-Zárate et al., 2011; Medina-Estrada et al., 2015). In this work, we show evidence that this response is regulated by global and specific histone marks on the promoter region of IIR genes and the expression of regulatory miRNAs.

Several reports have shown the role of epigenetic regulation in mammary gland function. For example, the levels of DNA methylation are important for α-casein gene expression (Singh et al., 2010), and an increase in this mark has been reported in peripheral blood lymphocytes from cows with mastitis (Song et al., 2016), as well as in the histone H3K27me3 mark (He et al., 2016). Besides, combined hormones bPRL and 17β-estradiol regulate H3 histone marks during *S. aureus* infection (Salgado-Lora et al., 2020). In this work, bPRL (24 h) and *S. aureus* reduced H3 global acetylation of bMECs (Figure 1B), which is related to chromatin compaction that make difficult the access of the transcriptional machinery. This result agrees with the down-regulation of pro-inflammatory genes *TNF-α, IL-1β*, and *IL-8* in *S. aureus*-challenged bMECs and treated with bPRL previously reported (Medina-Estrada et al., 2015). Also, the H3K9ac mark was increased by *S. aureus* in bMECs, but it was reduced in the presence of bPRL (Figure 1C). These results agree with the up-regulation of this mark by *S. aureus* reported in a murine mastitis model (Modak et al., 2014). Noteworthy, the H3K9me2 mark was increased by *S. aureus* and bPRL in bMECs (Figure 1D), and it inversely correlated with the H3K9ac mark, which also agrees with light lysine demethylases (KDMs) activity reduction (Figure 2C). Interestingly, the H3K9ac mark reduction and H3K9me2 induction in bMECs treated with bPRL and challenged coincides with the increase in the HDACs activity (Figures 2A,B), which suggests differential regulation of these enzymes. A similar effect was reported by Báez-Magaña et al. (2020), who showed that *S. aureus* induced the HDACs activity in bMECs, which was enhanced by the antimicrobial peptide γ-thionin. However, an opposite effect was reported in bMECs challenged with *S. aureus* and treated with the combined hormones bPRL and 17β-estradiol, in this case, the histone acetylation was increased, but HDACs activity was down-regulated or inhibited (Salgado-Lora et al., 2020). Possibly, 17β-estradiol could be inhibiting the effect of bPRL; however, the cross-linking between both hormone signaling pathways needs further research (Rasmussen et al., 2010).

From those mentioned above, we hypothesized that prolactin could regulate the gene expression of elements of IIR in bMECs through the induction of specific epigenetic marks such as H3K9ac and H3K9me2. ChIP assays showed that bPRL favors the enrichment of the H3K9ac mark in the promoter region of *IL-1*β and *IL-10* genes (Figure 3A), which suggests chromatin decompaction that allows the transcriptional machinery access; however, *S. aureus* reduced this mark (Figure 3A), suggesting that the chromatin has been condensed. These results agree with a previous report of our group where it was shown that bPRL increased both gene expression and secretion of IL-1β and induced the expression of IL-10, while *S. aureus* downregulated the expression of both genes (Medina-Estrada et al., 2015). Similar findings were reported by Modak et al. (2014) in a murine mastitis model. Also, we observed an inverse relationship between the enrichment of the H3K9ac mark and the H3K9me2 mark in the promoter region of *IL-1β* and *IL-10* genes (Figures 3A,B) under the conditions mentioned above. Similar behavior was observed for the *BNBD10* gene concerning the H3K9ac mark (Figure 3A), which was consistent with this gene’s expression level (personal communication). However, for this gene, even though there was a significant attenuation of the enrichment of the H3K9me2 mark (Figure 3B) in all of the conditions evaluated, suggesting the participation of other repressor marks in the negative regulation for the expression of *BNBD10* gene. In this sense, an opposite effect was observed on the *LAP* gene. bMECs only challenged with *S. aureus* showed significant enrichment of the H3K9me2 mark in the promoter region of this gene, which was attenuated in the presence of the hormone (Figure 3B), while there was no significant enrichment of the H3K9ac mark or inverse correlation concerning the H3K9me2 (Figure 3A); thus, probably the expression of this gene may be regulated for another epigenetic mark such as H3K14ac, H3K27ac, or H3K4me3, which are involved in the relaxing of chromatin and positive expression of genes (Modak et al., 2014). Further research is necessary to respond to these issues.

Previously, our group reported that *S. aureus* induced both the gene expression and the secretion of TNF-α in bMECs, and this expression was downregulated by bPRL (Medina-Estrada et al., 2015). This is consistent with our results because the H3K9ac mark was increased on the promoter region of the *TNF-α* gene in bMECs only challenged and was reduced by bPRL (Figure 3A). Also, an inverse relationship was observed between the enrichment of the H3K9ac mark and the repressor H3K9me2 mark on the promoter region of the *TNF-α* gene (Figure 3B). A similar effect was observed in the promoter region of the *IL-8* gene concerning H3K9me2 (Figure 3B), except that an inverse correlation concerning the H3K9me2 mark only was observed in cells treated or not treated with the hormone and challenged with *S. aureus* (Figure 3B). To our knowledge, this is the first report that correlates epigenetics marks in the regulation of IIR by the lactogenic hormone bPRL in bMECs challenged with *S. aureus*.

On the other hand, small non-coding RNA molecules such as miRNAs are involved in regulating many physiologic processes; their role has been reported particularly in regulating the immune response during infectious diseases (Hirschberger et al., 2018; Nejad et al., 2018). To evaluate other epigenetics mechanisms modulated by bPRL, the expression of miRNAs involved in the regulation of IIR genes by *S. aureus* such as mir-21a, miR-30b, Let-7a-5p, miR-155, miR-23a, miR-7863, miR-144, miR-146a, and mi-451 was analyzed (Jin et al., 2014). Let-7a-5p, miR-21a, and mir-30b have a key role in the regulation of expression of IIR genes such as IL-6, IL-1β, and TNF-α through the direct interaction with elements downstream of the signaling pathway of Toll-like receptors (TLRs) and it has been reported that their overexpression promotes the internalization and persistence of intracellular bacteria in the cells host (Zhou et al., 2018). We showed that Let-7a-5p, miR-21a, and mir-30b were up-regulated in bMECs challenged with *S. aureus* and down-regulated significantly in the presence of bPRL (Figures 4A–C). This is in agreement with Xu et al. (2021), who demonstrated that the pro-inflammatory response induced by bacterial lipopolysaccharide (LPS) is alleviated when miR-30b is silenced (Xu et al., 2021). In addition, Corsetti et al. (2018) reported that inhibition of expression of miR-21a induced the downregulation of TNF-α and promoted bacterial control in the host cell (Corsetti et al., 2018). In contrast to our results, Magryś and Bogut (2021) reported that a small variant colony (SVC) of *Staphylococcus epidermidis* down-regulated the expression of Let-7a-5p changing the pro-inflammatory profile to an anti-inflammatory environment that favors its intracellular persistence in macrophages THP-1. In this sense, Let-7a-5p could be key in the immune response of bMECs during *S. aureus* infection because the upregulation of this miRNA coincides with the downregulation of expression of IL-6 (Medina-Estrada et al., 2015; Zaatout et al., 2020). Further investigation is necessary to identify the molecular targets of miR-21a, and mir-30b and the key role in the immune response in bMECs challenged with *S. aureus* and treated or not with bPRL. On the other hand, miR-155 is the master regulator of pro-inflammatory genes, which are up-regulated through the signaling pathway of TLRs; this miRNA targets the IKKβ kinase, which is involved in the modulation of translocation of the transcription factor nuclear factor κ B (NF-κ B) to nuclei and promotes the expression of pro-inflammatory mediators (Zhou et al., 2018). In this sense, the interaction IKKβ k-miR-155 promotes in bacterial infections the escape to immune response mounted for the host through the downregulation of the expression of pro-inflammatory genes (Zhou et al., 2018). In this study, we showed that *S. aureus* promotes a significant upregulation of miR-155 in bMECs. This expression was attenuated in the presence of bPRL (Figure 4D). This supports the fact that miR-155 plays a key role in the success of the establishment and persistence of bacterial infection. Moreover, it has been reported that overexpression of miR-155 induces the expression and production of TNF-α in mycobacterial infection (Behrouzi et al., 2019). This is consistent with our results, where we showed an H3K9ac mark increased in the promotor gene of TNF-α (Figure 3A), which promotes the transcription of this gene and protein secretion (Medina-Estrada et al., 2015). Interestingly, the presence of bPRL attenuated the expression of miR-155 and coincides with the reduction of the H3K9ac mark in the promoter region of TNF-α and the reduction of gene expression and protein secretion (Medina-Estrada et al., 2015). A similar effect was reported by Rajaram et al. (2011), who demonstrated that lipomannan, a component of the cellular wall of *Mycobacterium tuberculosis*, blocks TNF-α synthesis mediated by mir-155 (Rajaram et al., 2011). In this sense, bPRL could be key for the downregulation of TNF-α through H3K9ac and miR-155. miR-23a is also a miRNA that regulates the inflammatory response by inhibiting the translocation of NF-κ B to nuclei and targeting PI3K kinase (Zhou et al., 2018). Recently, Cai et al. (2021) reported that short exposition of lipoteichoic acid (LTA), a component of the cellular wall of *S. aureus*, induced the overexpression of miR-23a, which targeted the PI3K kinase-TLR2-MyD88 axis and downregulated the pro-inflammatory response in the cell line MAC-T (bovine mammary epithelial cells), while the inhibition of expression of miR-23a or long exposition of LTA reversed this effect (Cai et al., 2021). Our results showed a similar effect in bMECs treated only with bPRL; we observed a significant increment of expression of miR-23a, but this expression was downregulated in the presence of the bacteria (Figure 4F). Possibly, short periods of the exposition of bMECs with *S. aureus* are sufficient to decrease the protective effect of bPRL through miR-23a-TLR2-MyD88-IP3K kinase. However, the regulatory mechanism of miR-23 related to the immune response is still poorly understood. We detected a significative level of expression of miR-7863 in bMECs challenged with *S. aureus*, which was down-regulated in the presence of bPRL (Figure 4E). This agrees with Luoreng et al. (2018). They reported through transcriptional analysis the expression of miR-7863 only in mastitis caused by *S. aureus* or *E. coli*. In this sense, miR-7863 could be a biomarker of bovine mastitis. Moreover, given that bPRL down-regulated the expression of miR-7863, it could be interesting to investigate the target of this miRNA and the molecular mechanism involved in the immune response during bacterial infection. Finally, even though miR-451, miR-144, and miR-146a are important regulators of the pro-inflammatory during bacterial infections (Zhou et al., 2018), we did not observe any changes in the expression level of these miRNAs in our model of study (Figures 4G–I). This suggests that miR-451, miR-144, and miR-146a are not key in regulating the immune response of bMECs during *S. aureus* challenge.

## Conclusion

Bovine prolactin regulates the IIR of bMECs through modifications of H3K9ac and H3K9me2 marks and the expression of miRNAs. In general, we observed that bPRL induced an anti-inflammatory profile in bMECs through the regulation of epigenetic marks in promotor genes of key cytokines and in the induction of miRNAs that block the pro-inflammatory cytokine production, reverting the inflammatory response induced by *S. aureus*. To our knowledge, this is the first report that correlates epigenetic mechanisms in regulating elements of IIR modulated by lactogenic hormone bPRL in bMECs challenged with *S. aureus*.

## Data availability statement

The raw data supporting the conclusions of this article will be made available by the authors, without undue reservation.

## Author contributions

MB-M, MS-L, JL-M, and AO-Z conceptualized and designed the experiments and analyzed the data. MB-M and MS-L performed the experiments. MB-M, JL-M, and AO-Z wrote the manuscript. All authors read and approved the final manuscript.

## Footnotes

1
http://genomics.dote.hu:8080/mirnadesigntool/
